# Engaging Community College Students in Publishable Research

**DOI:** 10.3389/fpsyg.2019.00882

**Published:** 2019-04-23

**Authors:** Russell J. Frohardt

**Affiliations:** Dean for Academic Success, Northwest Vista College, San Antonio, TX, United States

**Keywords:** extramural funding, guided pathway, active learning, community college, experiential learning, co-curricular activities, STEM - science technology engineering mathematics, hispanic serving institution (HSI)

Community colleges in the United States of America provide their surrounding communities with completely open access, quality higher education opportunities at a fraction of the cost for a 4-year college or university. The average annual cost (tuition and fees in district) for community colleges is $3,570, compared to $9,970 for in-state students to attend a 4-year public college (College Board, 2017). According to the American Association of Community Colleges (AACC), 7.1 million students enrolled in community colleges for credit in the Fall of 2016, with another 5 million students enrolled for non-credit courses (American Association of Community Colleges, 2018a). To put these enrollment figures in perspective, 41% of all undergraduates in the U.S. (National Center for Educational Statistics, 2018) are being introduced to higher education by faculty members at a community college. The open access provided by community colleges confronts inequities observed in other sectors of higher education. For example, 52% of Hispanics, 43% of Blacks, 56% of Native Americans, and 40% of Asians and Pacific Islanders who enrolled in college in Fall 2015 attended community colleges (National Center for Educational Statistics, 2018). Thus, community colleges provide the opportunity to engage our most underrepresented students in educational and research activities.

As an academic administrator at a community college (Northwest Vista College; NVC), one of my main goals is to help students succeed in their academic journey—and their subsequent careers—by providing the highest quality experiential learning opportunities possible. An important type of experiential learning, especially for students in science fields such as psychology, is early exposure to research opportunities with passionate faculty scholars. Yes, I said passionate community college faculty **scholars**. While the teaching load is often heavy, and promotion and tenure requirements at community colleges focus less on scholarship than other higher education sectors, we have active researchers within our faculty who have found innovative ways to involve students in their scholarship. In this article, I explore several ways that we can best support the process of engaging undergraduates in publishable research at community colleges.

## Funding Opportunities

My personal journey to become a scientist began with the opportunity to do research in a behavioral neuroscience lab and then present a poster of my work at the annual meeting of the Society for Neuroscience. I won't say how many years ago that was, but let's just say lots of spray glue was involved. That experience offered me the opportunity to speak with peers, mentors, and scientists from around the world about data and scientific methods. As a first-generation college student, I was fortunate to be afforded those opportunities through scholarships, institutional support, and extramural funding at an R1 (Research 1 or highest research activity Carnegie classification) university. We can provide the same, or similar, experiences for students at Masters-granting comprehensive universities, liberal arts colleges, and universities… and community colleges.

Most professional organizations affiliated with psychology and neuroscience have scholarships, discounts, and other funding support for students and faculty to present at local, regional, and national meetings and to conduct original research in a variety of topic areas. For example, the American Psychological Association's (2018a,b) Grants, Awards and Funding page boasts more than 600 scholarships, grants, and awards sponsored by APA and other psychology-related organizations. Some opportunities are restricted to graduate students and postdoctoral fellows, but many of them are available to undergraduates and faculty members at any institute of higher education. Special attention should be paid to awards for military veterans and Division 19 (Society for Military Psychology), women and Division 35 (Society for the Psychology of Women), underrepresented groups, educators, and Division 2 (Society for the Teaching of Psychology). The Association for Psychological Science (2018a) also has grants and awards available, as well as links (Association for Psychological Science, 2018b) to other funding sources and psychological science organizations including the Society for Neuroscience (2018). Honor societies also support research and travel to conferences. Psi Beta, the national honor society for psychology at community colleges, has travel and research scholarships available to promote research conducted at community colleges at the annual APA conference (Psi Beta, 2019).

Some extramural funding opportunities are specifically designed to aid undergraduate research, STEM (science, technology, engineering, and math) fields, underrepresented student populations, and partnerships between community colleges and 4-year colleges, local school districts, government and industry, clinical practices, and other community groups. For example, the National Science Foundation's (2018) *Improving Undergraduate STEM Education: Hispanic-Serving Institutions (HSI) Program* requests proposals to improve STEM education at HSIs by bridging the transition between community colleges and 4-year institutions.

Grants from NSF also support research initiatives and conferences specific to community colleges through organizations such as the Community College Undergraduate Research Initiative (2019). Other funding opportunities, such as the U. S. Department of Education's (2018) Developing Hispanic-Serving Institutions (DHSI)-Title V program and the Howard Hughes Medical Institute (2018) encourage collaborations to further science and science education between colleges and universities, as well as K-12 schools and community organizations. Perhaps more important than providing the financial support that enables research and publication, these programs help students, particularly those coming from economically disadvantaged backgrounds, think of themselves as scientists that can make meaningful contributions to their discipline.

## Prioritizing Experiential Learning

Funding from professional organizations in psychology and neuroscience are generally limited and competitive. To open up research, presentation, and publication opportunities for all students, institutions of higher education need to create a culture of support and funding for students, faculty, and staff that engage in these endeavors. Curricular and co-curricular activities can expose students to both the knowledge and skills necessary to produce publishable research in psychological science. Educators at all levels in higher education are recognizing the benefits of active learning inside and outside of the traditional classroom, getting away from the “sage on the stage” model of teaching (e.g., Bowen, 2012). Some techniques include the flipped classroom (i.e., delivering content outside of class via online content and readings, then doing activities, and “homework” in class), cooperative learning (i.e., small groups of students working on a common task), and problem-based learning (i.e., students experience a subject by solving an open-ended problem found in designated material; for a review, see Davidson et al., 2014). Such active forms of learning lay the groundwork for the types of inquiry required to conduct publishable research.

When traditional laboratory experiences are not a reasonable option, embedding scientific reasoning, and critical thinking modules into introductory psychology courses can significantly improve the skills important for conducting publishable research. For example, Stevens et al. (2016) found that students showed significant gains in scientific reasoning after experiencing carefully planned lectures and discussion modules designed to promote scientific reasoning and critical thinking. Such curricular enhancements, paired with experiential learning activities, promote faculty and student scholarship.

## Scaffolding Community College Students Toward Publishable Research

Many community colleges throughout the U.S. are attempting to enhance experiential learning opportunities by adopting a holistic model for supporting student success—the guided pathways model (Bailey et al., 2015; American Association of Community Colleges, 2018b). The model's first two dimensions clarify paths to student end goals and help students choose and enter a pathway (American Association of Community Colleges, 2018b; Community College Research Center and American Association of Community Colleges, 2018). The third and fourth dimensions help students stay on a path and ensure that students are learning. One of the essential practices under the third dimension is to embed academic and non-academic supports to promote student learning and persistence. Dimension 4 integrates applied learning experiences—such as research experiences—to enhance student success in courses across programs of study. Although the main goal of the pathways approach is to ensure that students complete an educational path and earn a credential, the model has the added benefits of exposing students to scientific inquiry and hands-on activities early in their careers, laying the foundation for success.

Within NVC's community college system, district leadership (board and administration) has funded many experiential and applied components of the guided pathways model. These resources and emphasis on experiential learning encourage our faculty to design new ways to engage students in original research and enable students and faculty to present their scholarly projects. Further, we invite speakers to campus for talks about their scientific and applied projects, and develop internal events (e.g., Psychology Day) and community outreach events (e.g., Sci-Tober, a showcase of various science disciplines to current and incoming students).

At this point, you may be convinced that community colleges can lay the groundwork for developing the skills needed to produce publishable research at a 4-year institution. Yet, you may be skeptical that community college students can actually produce publishable research. They can and they do. For example, Dr. William Altman of SUNY Broome Community College teaches a psychology research capstone where students “perform original research in psychology, to produce professional publications or presentations” (Altman, 1995). The prerequisites include Introductory Psychology and College Writing—not statistics nor research methods. Although some disciplinary research would require students to have developed additional content, statistical, or laboratory skills (e.g., neuroscience), community college students can produce publishable research under the mentorship of innovative and dedicated professors.

## Encouraging and Recognizing Strong Mentorship

Kimberly Bress, a recent winner of the Barry M. Goldwater Scholarship (a prestigious award for sophomores and juniors planning on research careers in STEM fields), offered one piece of advice to undergraduates who are thinking about doing lab research: “…find a good mentor—someone who answers your questions and really engages you in the research process.” (Society for Neuroscience, 2017). Don Lucas, NVC's Psychology Discipline Coordinator, described his process for engaging students in research in a recent newsletter article for the Southwestern Psychological Association (Lucas, 2018). He recruits students who possess drive and curiosity—not experience and a strong GPA. After completing ethics training, teams of students create testable hypotheses. Lucas then teaches students how to conduct literature reviews and read scientific articles. Students collect their own data and present their findings. Lucas and his colleagues regularly publish and present with undergraduates, and might engage even more students with the implementation of the guided pathways model described above.

## Conclusion

Forty percent of all first-time freshmen attend community college in the U.S (National Center for Educational Statistics, 2018). To engage more undergraduates at community colleges in publishable research, we need to increase resources, build research culture (perhaps embedded into the curriculum), and support faculty mentors. Increasing those resources may mean lobbying the local community and legislature to support more bond initiatives and state funding, convincing college administrators to provide more development opportunities and course release, and pursuing external grants. Building a research culture can grow from faculty and student journal clubs, co-teaching, college awards, hosting student research conferences, attending professional conferences, and co-curricular programming. Finally, it is important to encourage and reward faculty who mentor students. Community college faculty and staff are passionate about changing our students' lives. Building a foundation of scientific inquiry and writing in psychology at community colleges prepares students to conduct and publish original research while attending community college or beyond.

## Author Contributions

The author confirms being the sole contributor of this work and has approved it for publication.

### Conflict of Interest Statement

The author declares that the research was conducted in the absence of any commercial or financial relationships that could be construed as a potential conflict of interest.

## References

[B1] Altman W. S. (1995). Psychology Research Capstone (Writing Emphasis) – PSY 295. [Syllabus]. Retrieved from: https://jillshultz.com/williamaltman/Courses/Syllabi%20and%20Presentation%20Schedules/PSY%20295%20Syllabus.pdf (accessed March 22, 2019).

[B2] American Association of Community Colleges (2018a). Membership Database. Retrieved from: https://www.aacc.nche.edu/research-trends/fast-facts/

[B3] American Association of Community Colleges (2018b). AACC Pathways Project. Retrieved from: https://www.aacc.nche.edu/programs/aacc-pathways-project/

[B4] American Psychological Association (2018a). Grants, Awards and Funding Page. Retrieved from: https://www.apa.org/about/awards/index.aspx

[B5] American Psychological Association (2018b). Regional Psychological Associations. Retrieved from: https://www.apa.org/about/apa/organizations/regionals aspx

[B6] Association for Psychological Science (2018a). Grants, Awards, and Symposia. Retrieved from: https://www.psychologicalscience.org/members/grants-awards-and-symposia

[B7] Association for Psychological Science (2018b). Policy Links. Retrieved from: https://www.psychologicalscience.org/policy/policy-links

[B8] Bailey T. R. Smith Jaggars S. Jenkis D. (2015). Redesigning America's Community Colleges: A Clearer Path to Student Success. Cambridge, MA: Harvard University Press.

[B9] Bowen J. A. (2012). Teaching Naked: How Moving Technology Out of Your College Classroom Will Improve Student Learning. San Francisco, CA: Jossey-Bass.

[B10] College Board (2017). Trends in College Pricing: 2017. Retrieved from:https://trends.collegeboard.org/sites/default/files/2017-trends-in-college- pricing_0.pdf

[B11] Community College Research Center American Association of Community Colleges (2018). Pathways Model Description. Retrieved from: https://www.aacc.nche.edu/wp-content/uploads/2018/01/12PathwaysModelDescriptionFinal1616.pdf

[B12] Community College Undergraduate Research Initiative (2019). Retrieved from: https://www.ccuri.org/

[B13] Davidson N. Major C. H. Michaelsen L. K. (2014). Small-group learning in higher education—cooperative, collaborative, problem-based, and team-based learning: an introduction by the guest editors. J. Excell. College Teach. 25, 1–6.

[B14] Howard Hughes Medical Institute (2018). Retrieved from: https://www.hhmi.org/programs

[B15] Lucas D. (2018). Community college students conducting scientific research. Southwestern Psychol. 11:3.

[B16] National Center for Educational Statistics (2018). IPEDS Fall 2016 Enrollment Survey [AACC Analysis]. Retrieved from: https://nces.ed.gov/pubs2018/2018002.pdf

[B17] National Science Foundation (2018). Improving Undergraduate STEM Education: Hispanic-Serving Institutions Program. Retrieved from: https://nsf.gov/ehr/HSIProgramPlan.jsp

[B18] Psi Beta (2019). Awards Overview and Deadlines. Retrieved from: https://psibeta.org/awards/current-deadlines/

[B19] Society for Neuroscience (2017). Neuronline. Career Advice, An Undergraduate Research Story: Part One. Retrieved from: https://neuronline.sfn.org/Articles/Career-Advice/2017/An-Undergraduate-Research-Story-Part-One

[B20] Society for Neuroscience (2018). Retrieved from: https://www.sfn.org

[B21] Stevens C. Witkow M. R. Smelt B. (2016). Strengthening scientific reasoning skills in introductory psychology: evidence from community college and liberal arts classrooms. Scholar. Teach. Learn. Psychol. 2, 245–260. 10.1037/stl0000070

[B22] U. S Department of Education (2018). Developing Hispanic-Serving Institutions-Title V. Retrieved from: https://www2.ed.gov/programs/idueshsi/index.html

